# X-Linked Retinoschisis in Females in a Consanguineous Family: A Rare Entity

**DOI:** 10.4274/tjo.galenos.2020.42815

**Published:** 2020-08-26

**Authors:** Mehmet Önen, Kürşat Zor, Erkut Küçük, Gamze Yıldırım

**Affiliations:** 1 Ankara City Hospital, Clinic of Ophthalmology, Ankara, Turkey; 2 Niğde Ömer Halisdemir University Faculty of Medicine, Department of Ophthalmology, Niğde, Turkey; 3 Niğde Ömer Halisdemir Training and Research Hospital, Clinic of Ophthalmology, Niğde, Turkey

**Keywords:** X-linked retinoschisis, consanguineous marriage, cystoid macular edema, macular atrophy, optical coherence tomography

## Abstract

X-linked juvenile retinoschisis (XLRS) is a disease considered characteristic for males. In this study we report a consanguineous family in which 3 daughters were diagnosed with XLRS. Typical signs of XLRS were detected in 2 girls, aged 4 and 15. Fundoscopic examination of the father and the oldest daughter (age 17) revealed bilateral atrophic macula and retinal thinning. Although rare and considered characteristic for males, XLRS can be seen in females in Middle-East countries that have a high rate of consanguineous marriage. It can be overlooked by ophthalmologists and these patients may be misdiagnosed.

## Introduction

Although X-linked retinoschisis (XLRS) is reported as rare in the literature, XLRS is the most common cause of juvenile macular degeneration.^1,2^ Diagnosis can be established through a fundus examination and optical coherence tomography (OCT), but electroretinography (ERG) is required for a definitive diagnosis.^1,3,4^

In this study we present the findings of a family with consanguineous marriage from the Central Anatolia region of Turkey in which the father and 3 daughters were affected by XLRS. The family consisted of the father and 3 daughters with XLRS, and the mother and another daughter who had normal ocular examination findings. The diagnosis of this rare disease is quite difficult even in the typical male patients and we want to emphasize the rare possibility of XLRS in female patients, especially in countries with high frequency of consanguineous marriage.

## Case Report

**Figure 1:**
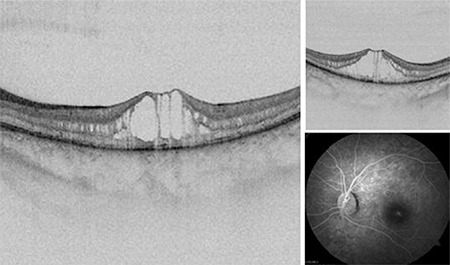
Right and left eye OCT images of the 15-year-old patient show the space that caused splitting of the greater hyporeflective neurosensorial retina, and many tiny perifoveal hyporeflective spaces in the internal and external nuclear layers OCT: Optical coherence tomography

A 15-year-old girl presented to another center with symptoms of poor vision. Her vision was 20/40 in both eyes. She was diagnosed as having idiopathic cystoid macular edema (CME) and referred to our clinic for detailed examination. A space-causing split in the greater hyporeflective neurosensorial retina was detected on OCT imaging (Figure 1). Numerous tiny perifoveal hyporeflective spaces were detected in the internal and external nuclear layers, mostly in the internal nuclear layer. No fluorescein leakage was detected in fundus fluorescein angiography (FFA) imaging (Figure 1). Reduced b-wave amplitude was detected on ERG.

She was diagnosed as having foveomacular retinoschisis after ophthalmologic examination and imaging. Therefore, all of her family members (mother, father, and 3 sisters aged 4, 11, and 17) underwent a detailed eye examination including best-corrected visual acuity (VA) (using Early Treatment Diabetic Retinopathy Study charts), slit-lamp biomicroscopy of the anterior segment, intraocular pressure measurement using Goldmann applanation tonometry, and dilated fundoscopic examination. Ocular imaging techniques such as digital fundus photography and OCT were also performed. FFA was only performed for the 15-year-old patient who had retinoschisis. ERG was performed in accordance with the International Society of Clinical Electrophysiology of Vision guidelines.

**Figure 2:**
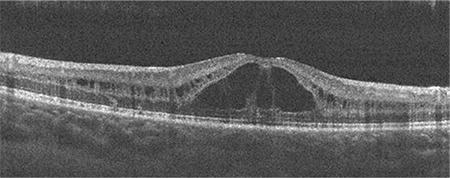
Right eye OCT image of the 4-year-old patient shows the space that caused splitting of the neurosensorial retina OCT: Optical coherence tomography

The 4-year-old daughter’s vision was 20/50. Her examination, imaging, and ERG findings were similar to those of her 15-year-old sister (Figure 2).

**Figure 3:**
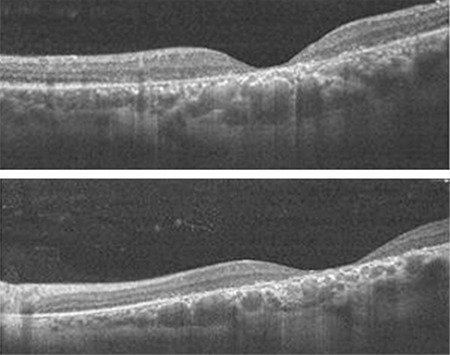
Right and left eye OCT images of the father show atrophy of the fovea OCT: Optical coherence tomography

The father, aged 42 years, had symptoms of low vision since childhood, but had never been diagnosed as having XLRS. His VA was 20/125 in the right eye and 20/100 in the left eye. Fundoscopy revealed atrophy in both maculae, and pigment epithelial change was observed. Retinal thinning and atrophy were detected in the macular area on OCT imaging (Figure 3).

**Figure 4:**
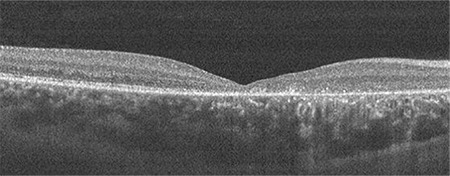
Right eye OCT image of the 17-year-old patient shows atrophy of the fovea OCT: Optical coherence tomography

The 17-year-old daughter had not previously consulted an ophthalmologist. Her bilateral vision was 20/100. Macular atrophy and pigment epithelial change were detected on fundoscopy. Retinal thinning was detected in the macular area on OCT imaging (Figure 4). Unexpectedly, peripheral retinoschisis was not detected in any of the XLRS patients in the family.

Although an a-wave typical for XLRS was detected in ERG in the father and the 17-year-old daughter, no attenuation of the b-wave was observed. This might be due to deterioration of the macular pigment epithelium in both patients.

No significant defect was detected in any of the family members on refraction examination.

In the mother and 11-year-old daughter, bilateral vision was 20/20, no ocular pathology was detected, and there were no abnormal findings in ERG.

## Discussion

XLRS has been reported in a limited number of studies in female children, particularly of consanguineous parents.^1,5,6^ In our study, we report a family with consanguineous marriage from Turkey in which the father and oldest daughter (age 17) had macular atrophy and 2 other daughters (age 4 and 15) had active foveal retinoschisis with no peripheral retinoschisis, which had been misdiagnosed as idiopathic macular edema. Although foveal retinoschisis is detected in almost all patients, schisis and cystic changes disappear in some patients in adulthood, and a nonspecific macular appearance develops. This nonspecific appearance might hinder accurate diagnosis.^7^ Researchers in the literature generally reported atrophy in individuals over the age of 30 years; however, we detected macular atrophy in the father, aged 42, and the 17-year-old daughter, and both showed retinal thinning in the foveal area on OCT.

The appearance of foveomacular retinoschisis may be confused with CME. However, FFA of patients with retinoschisis is often normal, or a slightly hyperfluorescent circular zone is observed in the edges of the macular area.^8^ The first member of the family was a 15-year-old daughter referred to our clinic with the diagnosis of CME. We detected no leakage suggesting CME on FFA of this patient, and we were unable to perform FFA in the 4-year-old daughter with retinoschisis. Goldmann-Favre syndrome, which is characterized by foveal schisis, is also important in the differential diagnosis. Autosomal recessive transmission, severe nyctalopia, pigmentary retinopathy, and attenuation of a- and b-waves in ERG are important for differentiation from XLRS.^2^

### Study Limitations

One of the limitations of our case was that we could not draw a family tree due to missing data about the ocular condition of the families of the mother and father. Another limitation was that we did not conduct molecular gene analysis, which could have contributed to the literature. Such a workup would certainly have yielded very precious information regarding the possibility of meaningful genotype-phenotype correlations in X-linked juvenile retinoschisis.

In conclusion, XLRS, which manifests with low vision in the first decade of life, is detected at a high frequency in countries with high prevalence of consanguineous marriage, and the importance of genetic counseling is growing in these countries. Taking this rare condition into consideration will prevent incorrect treatment for CME in these patients. These patients may be treated for misdiagnoses such as amblyopia, strabismus, hyperopia, and astigmatism. The correct diagnosis can prevent the application of inappropriate treatments, and informing the patients about the risk factors for development of retinal detachment and taking precautions may prevent the occurrence of complications like retinal detachment. In addition, early diagnosis of individuals with XLRS will become more important because *RS1* (retinoschisis 1) gene therapy outcomes in animal models are promising.
